# Massive splenomegaly due to splenic marginal zone B‐cell lymphoma

**DOI:** 10.1002/jgf2.413

**Published:** 2020-12-22

**Authors:** Kiyoshi Shikino, Masatomi Ikusaka

**Affiliations:** ^1^ Department of General Medicine Chiba University Hospital Chiba Japan

**Keywords:** lymphoma, splenomegaly

## Abstract

A 73‐year‐old man presented with an abdominal mass that gradually swells over 3 months. He denied any subjective symptoms. Physical examination revealed massive enlargement of the spleen—the spleen had crossed the midline and its lower margin extended into the lower abdomen.

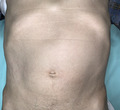

A 73‐year‐old man presented with an abdominal mass that gradually swells over 3 months. He denied any subjective symptoms. Physical examination revealed massive enlargement of the spleen—the spleen had crossed the midline and its lower margin extended into the lower abdomen (Figure 1). Laboratory data were as follows: hemoglobin level, 9.6 g/dL; white blood cell count, 5700/µL with 7% atypical lymphocytes; platelet count, 10.4 × 10^4^/µL; and soluble interleukin‐2 receptor level, 3975 U/mL. The liver function test results were normal. Computed tomography showed massive splenomegaly (Figure 2). Splenic biopsy revealed primary splenic marginal zone B‐cell lymphoma. The patient underwent a successful rituximab monotherapy and recovered.

**FIGURE 1:**
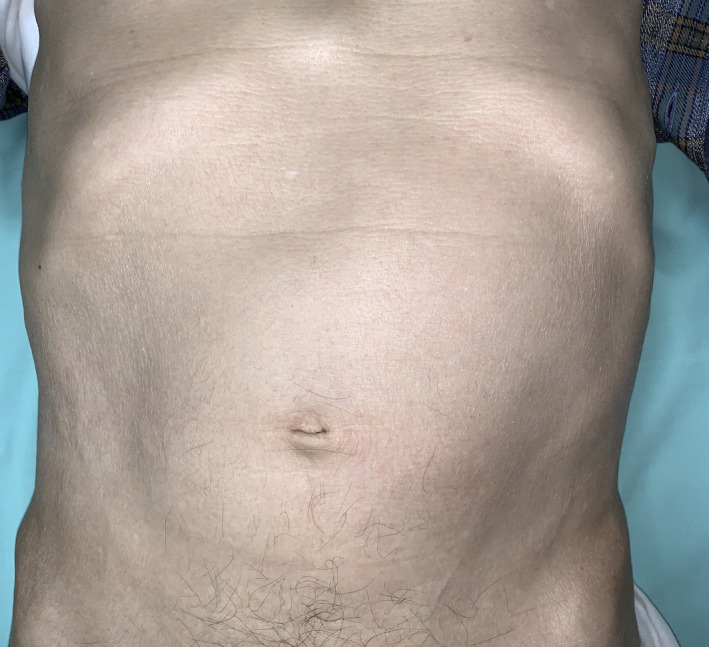
The spleen crossing the midline and its lower margin extending into the lower abdomen

**FIGURE 2:**
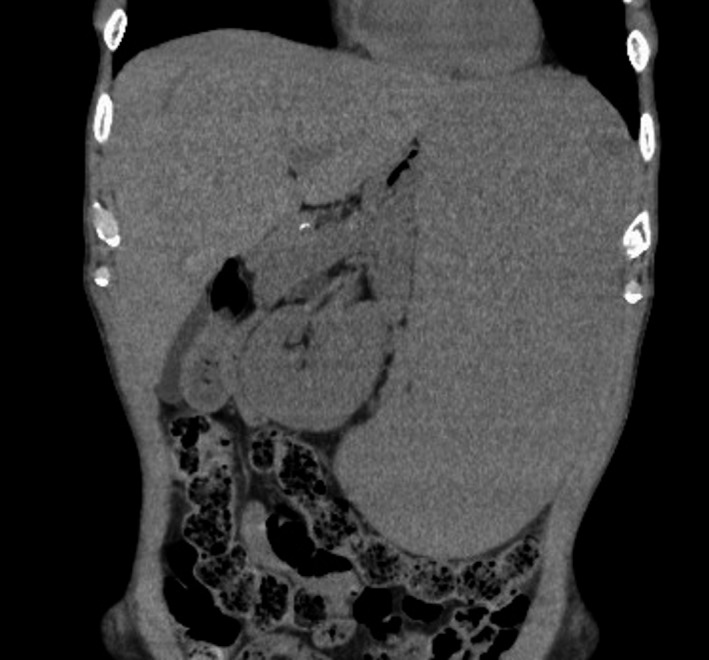
Computed tomography showed massive splenomegaly

Splenic marginal zone B‐cell lymphoma occurs in <2% of non‐Hodgkin's lymphomas and presents with splenomegaly or dull abdominal pain.^1^ Splenomegaly is “massive” when the spleen is palpable >8 cm below the costal margin.^2^ The differential diagnosis of splenomegaly, especially when massive, includes lymphomas, chronic myeloid leukemia, hairy cell leukemia, polycythemia vera, sarcoidosis, Gaucher disease, and infectious diseases.^3^ The recognized therapeutic options for splenic marginal zone B‐cell lymphoma are splenectomy, chemotherapy, rituximab alone, or rituximab plus chemotherapy.^4^

## CONFLICT OF INTEREST

None.

## AUTHOR CONTRIBUTIONS

All authors had access to the data and a role in writing the manuscript.

## INFORMED CONSENT

We have obtained the consent of the patient for publication.
